# A review of the pathogenesis of epilepsy based on the microbiota-gut-brain-axis theory

**DOI:** 10.3389/fnmol.2024.1454780

**Published:** 2024-10-03

**Authors:** Wentao Yang, Hua Cui, Chaojie Wang, Xuan Wang, Ciai Yan, Weiping Cheng

**Affiliations:** ^1^Department of Fist Clinical Medical College, Heilongjiang University of Chinese Medicine, Harbin, China; ^2^First Affiliated Hospital, Heilongjiang University of Chinese Medicine, Harbin, China

**Keywords:** microbiota-gut-brain-axis, epilepsy, gut barrier, blood–brain barrier, pathological, physiological

## Abstract

The pathogenesis of epilepsy is related to the microbiota-gut-brain axis, but the mechanism has not been clarified. The microbiota-gut-brain axis is divided into the microbiota-gut-brain axis (upward pathways) and the brain-gut-microbiota axis (downward pathways) according to the direction of conduction. Gut microorganisms are involved in pathological and physiological processes in the human body and participate in epileptogenesis through neurological, immunological, endocrine, and metabolic pathways, as well as through the gut barrier and blood brain barrier mediated upward pathways. After epilepsy, the downward pathway mediated by the HPA axis and autonomic nerves triggers “leaky brain “and “leaky gut,” resulting in the formation of microbial structures and enterobacterial metabolites associated with epileptogenicity, re-initiating seizures via the upward pathway. Characteristic changes in microbial and metabolic pathways in the gut of epileptic patients provide new targets for clinical prevention and treatment of epilepsy through the upward pathway. Based on these changes, this review further redescribes the pathogenesis of epilepsy and provides a new direction for its prevention and treatment.

## 1 Introduction

Epilepsy (Wirrell et al., 2022) is an episodic neurological disorder in which nerve cells in the brain hypersynchronize abnormal discharges, causing recurrent and transient brain dysfunction. Approximately 50 million people worldwide are currently living with epilepsy, and approximately 4 million new cases are diagnosed each year (Leonardi et al., 2024). Frequent seizures and/or epileptiform discharges cause irreversible damage to the brain (Bastos and Cross, 2020), resulting in progressive dysfunction or degeneration of cognitive, linguistic, sensory, motor and behavioral aspects of the patient, imposing a heavy burden on the individual, family and society. Despite the proliferation of epilepsy treatments in recent years, such as surgery, ketogenic diet, and deep brain stimulation, the etiology of epilepsy in two-thirds of patients has not been thoroughly elucidated (Duan et al., 2022; Serino et al., 2019), especially with the uncontrollable seizures in 20–30% of epileptic seizures as well as the prevalence of behavioral toxicities of antiepileptic drugs; therefore, epilepsy pathogenesis, treatment, and prevention remain a focus of current research in the medical community.

In recent years, many studies have found that the pathogenesis of central nervous system (CNS) diseases may be related to the gut microbiota (GM) (Ma et al., 2019; Amlerova et al., 2021; Zhang et al., 2023; Liu et al., 2020; Aljeradat et al., 2024). The GM contributes to the development and progression of neurological diseases from the bottom up by producing metabolites, stimulating nerves, activating immune pathways, etc. (Guo et al., 2024; Dicks, 2022). The brain characteristically alters the structure of the GM after the onset of the disease through neurological and endocrine pathways from the top down (Abdel-Haq et al., 2019; Wang and Kasper, 2014; Singh et al., 2016). The signaling pathway between the GM and the brain that influences brain-gut interactions through neurological, endocrine, immune, and substance metabolism mechanisms is known as the “Microbiota-gut-brain (MGB axis) axis “(Mayer et al., 2022). The signaling mechanism from the GM to the brain is designated as the upward pathway, while the signaling mechanism from the brain to the GM is called the downward pathway. The gut barrier and the blood–brain barrier (BBB) play crucial roles in ensuring gut and brain homeostasis, blocking the intrusion of risk factors into the MGB axis, and preventing the development of neurological diseases (Cryan et al., 2019; Socala et al., 2021). In addition, favorable therapeutic effects have been shown in drug-resistant epilepsy by reconstructing the epileptic host GM (Table 1) (Chen et al., 2023). This paper explores the pathogenesis of epilepsy based on the MGB axis by outlining the upward and downward pathways, providing new ideas for clinical diagnosis, treatment, and prevention of epilepsy.

**Table 1:** Focus on epilepsy in animal studies.

Assortment	Model group	Control groups	Induction of epilepsy modalities	Types of seizures	Molding time	Results	Conclusion	Samples	Methods	Limitations	References
Adult male Sprague Dawley (SD) rats	Antibiotic Group (Ciprofloxacin *n* = 33)	Control group (Ctrl *n* = 30), the same volume of normal saline (NS)	A single dose of pentylenetetrazole (PTZ)18 mg / mL	Acute seizures, Racine Scale, exhibit a score of 4	Antibiotic Group:ciprofloxacin monohydrochloride 100 mg/kg, per day for 14 days; Control Group: the same volume of normal saline (NS) by gavage for 14 days	↑*Akkermansia* and *Bacteroides*, ↓*Marvinbryantia, Oscillibacter* and *Ruminococcaceae_NK4A214_group,* ↓indole-3-propionic acid	Ciprofloxacin-induced seizure susceptibility is partially mediated by the gut microbiota and tryptophan-indole metabolism	Fecal samples, Serum	16S rRNA sequencing, Serum untargeted metabolomic analysis	The utilization of animal models in this study poses limitations when extrapolating the findings to human conditions	Zou et al. (2024)
Specific pathogen-free (SPF) C57BL/6 adult male mice	(1) immune-training group (1× LPS, dose of 500 μg/kg body weight),(2) immune-tolerant group (4× LPS, dose of 500 μg/kg body weight).n = 40 per group	Control group, the same volume of normal saline (NS), *n* = 40 per group	The kainic acid (KA) model, mice received injections of 0.05 μL KA (Sigma-Aldrich, 1 nmol/50 nL) into the right hippocampus area at a rate of 0.01 μL/min	Acute seizures, Racine Scale, exhibit a score of 4	LPS intervention for 4 days, followed by normal feeding for 2 weeks	*Ruminococcus* was the dominant flora	The intestinal flora can significantly change the immune state, thereby affecting neuronal excitability and improving the progress of epileptic episodes	Fecal samples	16S rRNA sequencing	The lack of behavioral data of over-excitement	Ding et al. (2022)
Adult female SD rats	(1) PTZ + saline: pentetrazol + saline (2) PTZ + VPA (Sodium valproate): sodium valproate (300 mg/kg) + pentetrazol (3) PTZ + P-37.5: sodium propionate (37.5 mg/kg) + pentetrazol (3) PTZ + P-50: sodium propionate (50 mg/kg) + pentetrazol PTZ + P-75: sodium propionate (75 mg/kg) + pentetrazol. *n* = 10 per group	Control group: no intervention, *n* = 10	PTZ at a dose of 37 mg/kg	Acute seizures, Racine Scale, exhibit a score of 4	Continued for 40 days	Improved ATP level and lessened 8-OHdG level, increase activities of the antioxidant enzymes (CAT, SOD, and GSH-Px)	Propionate might be a promising supplement in diet for protecting brain tissues from injury	Brain tissues	Gas chromatography (GC) analysis	/	Cheng et al. (2019)
Female and male SD rats,45 days old	(1) Sham stress, no microbiota transplant; (2) Stress, no microbiota transplant; (3) Sham-stressed recipients transplanted with microbiota from sham-stressed donors; (4) Stressed recipients transplanted with microbiota from sham-stressed donors; (5) Sham-stressed recipients transplanted with microbiota from stressed donors; (6) Stressed recipients transplanted with microbiota from stressed donors. Each consisting of 6 rats (3 of each sex)	/	A bipolar stimulating electrode kindling of the basolateral amygdala	With stage 4–5 seizures (ie, rearing, with or without falling) in response to 3 consecutive stimulations	Two 2-h long sessions per day, over 2 weeks	(2)(5)(6) ↑ADD, ↑ADT; (1)(3)(4) ↓ADD, ↓ADT.	Chronic stress alters gut microbiota to increase susceptibility to epilepsy	The duration of kindled seizures: the afterdischarge threshold (ADT) and afterdischarge duration (ADD)	Electroencephalogram (EEG) analysis	Microbiome sequencing in neither donors nor recipients, not investigate consequences of the stress-induced dysbiosis predisposing to epilepsy	Medel-Matus et al. (2018)
Male ICR mice, 5 weeks old	HFD (60 kcal % fat, 20 kcal % carbohydrate, and 20 kcal % protein), *n* = 14; ND and KA injection (ND + KA, n = 20), or HFD and KA injection (HFD + KA, n = 20)	Normal laboratory chow diet (ND, 18 kcal % fat, 58 kcal % carbohydrate, and 24 kcal % protein), n = 14	Intraperitoneal injection of 30 mg/kg KA	Acute seizures, Racine Scale, exhibit a score of 4	*Ad libitum* for 8 weeks and then received an intraperitoneal injection of 30 mg/kg KA	HFD-induced: the expression of calpain1↑, nuclear factor E2-related factor 2↑, heme oxygenase-1↑; the number of terminal deoxynucleotidyl transferase dUTP nick end labeling (TUNEL)-positive cells ↑	Obesity-induced systemic inflammation, neuroinflammation, ER stress, calcium overload, and oxidative stress may contribute to neuronal death after brain injury.	Serum and hippocampal tissues	TUNEL staining, immunohistochemistry, Immunofluorescence and western blot analysis	/	Kang et al. (2015)
Adult male CRH-GFP mice (C57Bl/6 background)	Vehicle, kainic acid (20 mg/kg), or pilocarpine (340 mg/kg). *n* = 7–13 mice per experimental group	/	Kainic acid (KA) 20 mg/kg by intraperitoneal injection; pilocarpine hydrochloride 320–340 mg/kg by intraperitoneal injection	Acute seizures, Racine Scale, exhibit a score of 4	30 min	↑ACTH levels, ↑corticosterone, ↓amplitude of sIPSPs, ↓KCC2, ↑NKCC1 after administration, positive correlation between the extent of epileptiform activity and corticosterone levels	Compromised GABAergic control of CRH neurons following an initial seizure event may cause hyperexcitability of the HPA axis and increase future seizure susceptibility	Blood samples, hippocampus	Western blot analysis, Immunofluorescence, Electroencephalogram (EEG)	/	O’Toole et al. (2014)

## 2 Upward pathway induction and prevention of epilepsy

**Figure 1:**
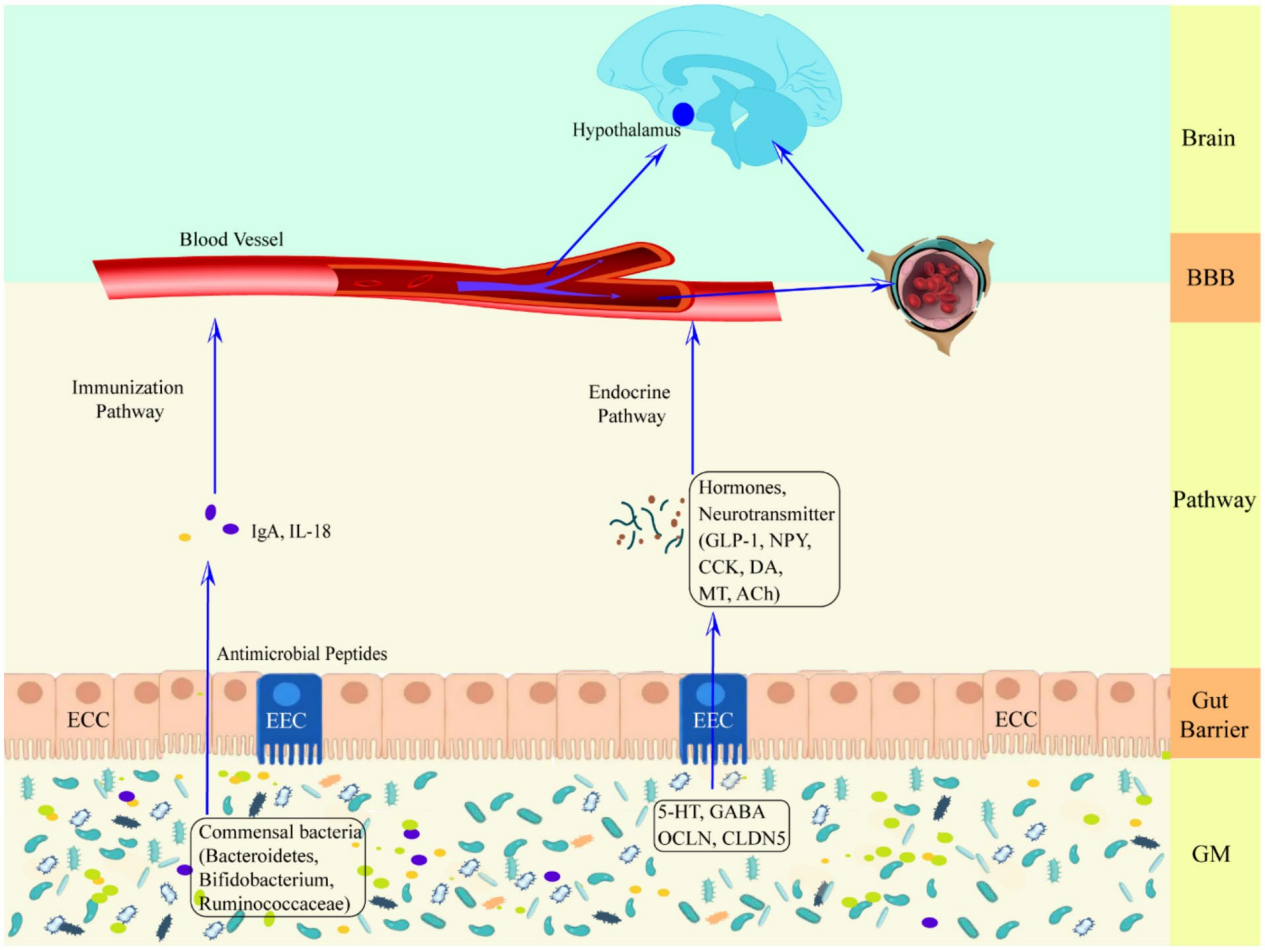
Schematic representation of the defense mechanism of the upward pathway “microbiota-gut-brain” through two barriers. Ach, acetylcholine; BBB, blood–brain barrier; CCK, cholecystokinin; CLDN5, claudin 5; DA, dopamine; ECC, enteroendocrine cell; EEC, enterochromaffin cell; GLP-1, glucagon-like peptide-1; GM, gut microbiota; HPA axis, hypothalamic–pituitary–adrenal axis; IgA, immunoglobulin A; IL-18, interleukin-18; MT, melatonin; NPY, neuropeptide Y; OCLN, occludin.

Healthy GM plays an essential role in host nutrient metabolism, drug metabolism, maintenance of the structural integrity of the gut mucosal barrier, immune regulation and defense against pathogens, etc (Wang and Wang, 2016). GM dysregulation and gut dysfunction can increase the risk of seizures (Table 2), Zou et al. (2024) found that ciprofloxacin treatment by tube feeding to alter GM and its metabolites increased susceptibility to pentylenetetrazol-induced seizures in rats, with a significant increase in the number of seizures and the mean duration of each seizure, but with a decrease in susceptibility after fecal transplantation. Riazi et al. (2008) found that colitis rats were highly susceptible to pentylenetetrazol-induced seizures, and it was closely related to the severity of gut inflammation. GM is involved in the pathogenesis of epilepsy through the action of “common information molecules and their corresponding receptors,” “metabolite changes” between the nervous, endocrine and immune systems, leading to structural and functional changes in the brain (see Figure 1). The endocrine and immune pathways differ from the direct neural pathway in that GM communication with the brain must be screened by two natural signaling barriers (the gut barrier and the BBB). Since gut microbes can modulate the permeability of both structures, the signaling from the gut to the brain is highly variable, depending on the state of the host. The relevant mechanisms are summarized below.

**Table 2:** Focus on epilepsy in human studies.

Epilepsy group and etiology	Control group	Seizure type	Treatment and duration	Results	Conclusion	Samples	Methods	Limitation	References
*N* = 94, Age: 4 months to 84 years, Etiology: unknown causes	*N* = 50, age and gender matched	Focal epilepsy	/	Thirteen (13.8%) patients, but none of the controls, had antibodies (*p* = 0.003)	Neuronal Autoimmune antibodies may be detected in patients with focal seizures regardless of prognosis	Plasma	Immunocytochemistry, Radioimmunoassay technique	Not including a control group with autoimmune disorders	Gozubatik-Celik et al. (2017)
*N* = 20 (14 males, 6 females), Age: 1.2 years to 10.3 years, Etiology: 5 patients were Dravet syndrome, 3 were West syndrome, 3 cases were Lennox–Gastaut syndrome, and other 9 children could not be classified as syndrome	/	Refractory epilepsy	KD therapy, duration: January 2015 and May 2016	↓alpha diversity, ↓Firmicutes, ↑Bacteroidetes, ↑Clostridiales, ↑Ruminococcaceae, ↑Rikenellaceae, ↑Lachnospiraceae, ↑Alistipes after KD therapy.	KD can reduce the species richness and diversity of intestinal microbiota	Fecal samples	16S rDNA high-throughput sequencing	/	Zhang et al. (2018)
*N* = 14 (epileptic infants,11 male and 3 female), Age: aged up to 3 years, Etiology: unknown, genetic, and structural	*N* = 30 (15 male and 15 female), Age: aged up to 3 years	Refractory epilepsy	KD therapy, duration:1 week	↓Proteobacteria, ↓Cronobacter, ↑Bacteroides, ↑Prevotella and ↑Bifidobacterium after KD therapy	KD could significantly modify symptoms of epilepsy and reshape the GM of epileptic infants	Fecal samples	16S rDNA sequencing	Difficult to unravel different microbes at the species or function level; Lack of a longer follow-up period; Lack of animal model validation	Xie et al. (2017)
*N* = 10, Age: 51 years to 71 years, Etiology: suspected autoimmune epilepsy (sAE)	*N* = 10, Age:50 years to 71 years, Etiology: noninflammatory CNS diseases	Late-onset seizures with an unknown etiology	/	The CSF levels of IL-6, IL-17, HMGB1, and CXCL12 higher than the control group, the CSF levels of IL-10, CXCL13 and BAFF no difference; The serum levels of HMGB1 and CXCL12 were elevated in the sAE group, the levels of IL-6, IL-10, IL-17, CXCL13, and BAFF no statistical difference	cytokines/chemokines may act as alternative biomarkers for diagnosis of sAE	Serum and CSF	Enzyme-linked immunosorbent assay (ELISA)	The relationship of the cytokines/chemokines and prognosis was not included because of the small sample size and the heterogeneity of etiology	Han et al. (2020)
Drug-resistant (DR) (*n* = 42), drug-sensitive (DS) (*n* = 49), Age:5 years to 55 years; Etiology: unknown, genetic, and structural	*N* = 65, families of the patients and had the same eating habits	Generalized epilepsy: DR 8 (19.0%), DS 16 (32.7%); Partial epilepsy: DR 4 (9.5%), DS 7 (14.3%); Multiple forms epilepsy: DR 30 (71.4%), DS 27 (55.1%)	/	DR: ↑rare flora; DS: similar with healthy controls; four seizures per year or fewer showed ↑Bifidobacteria, ↑Lactobacillus	Dysbiosis may be involved in the mechanism of drug-resistant epilepsy and restoring the gut microbial community may be a novel therapeutic method for drug-resistant epilepsy	Fecal samples	16S rDNA sequencing	/	Peng et al. (2018)
*N* = 41, Age: ages >18 years, Etiology: unknown, genetic, and structural	*N* = 30, families of the patients and had the same eating habits	All types of epilepsy were considered, including focal, general, structural, infectious, immune and hereditary	/	↑*Fusobacterium*, ↑*Megasphaera*, ↑*Alloprevotella*, ↑*Sutterella* in the epilepsy group at the genus level; *Fusobacterium* sp., *Fusobacterium mortiferum*, *Ruminococcus gnavus*, *Bacteroides fragilis* were significantly positively correlated with the occurrence of epilepsy	There were significant differences in the composition and functional pathways of gut flora between epilepsy patients and patient family members. The Fusobacterium may become a potential biomarker for the diagnosis of epilepsy	Fecal samples	16S ribosomal RNA sequencing	No direct evidence was provided that elevated levels of certain gut flora cause epilepsy; Small sample size	Dong et al. (2022)
*N* = 48, Age:19 years to 66 years, Etiology: unknown, genetic, and structural	*N* = 30, similar age and gender	Twenty-six patients (54%) with partial epilepsy, twenty-two patients (46%) with generalized epilepsy	/	IL-6, IL-17A, and IFN-γ were significantly elevated in epilepsy patients; Interictal IL17A concentration positively correlated with National Hospital Seizure Severity Scale (NHS3) scores and seizure frequency; Interictal IFN*γ* concentration was also showed positively correlation with seizure frequency	Postictal and interictal various inflammatory cytokines are elevated in plasma of active epilepsy patients. Furthermore, interictal IL-17A and IFN-γ may predict seizure severity	Venous blood samples	Enzyme-linked immunosorbent assay (ELISA)	/	Gao et al. (2017)

### 2.1 Neural pathways

**Figure 2:**
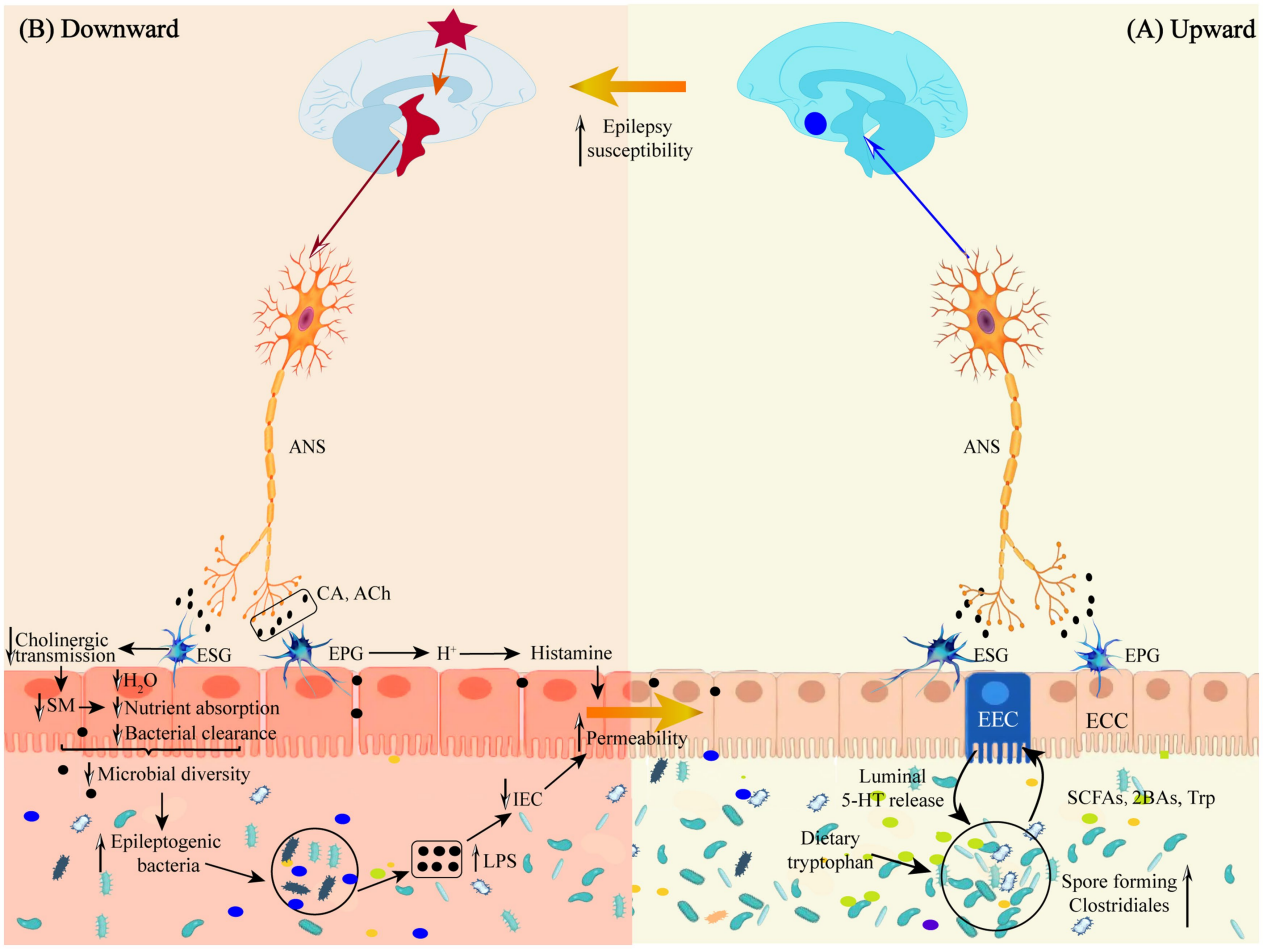
**(A)** Schematic representation of the defense mechanism of the “microbiota-gut-brain” direct neural pathway in the upward pathway. **(B)** Schematic diagram of the mechanism of injury in the downward pathway “epileptic brain-gut-microbiota” via the direct neural pathway. Ach, acetylcholine; ANS, autonomic nervous system; 2BAs, second bile acids; CA, catecholamine; ECC, enteroendocrine cell; EEC, enterochromaffin cell; EPG, Enteric Parasympathetic ganglion; ESG, Enteric sympathetic ganglion; IEC, intestinal epithelia cell; LPS, lipopolysaccharide; SCFAs, short-chain fatty acids; SM, smooth muscle; Trp, tryptophan.

A direct neural pathway exists between gut microbes and the brain that does not pass through a dynamic barrier and consists of neural pathways from the spinal enteric nervous system, autonomic nervous system, and vagus nervous system (Mayer et al., 2014; see Figure 2A).

The gut has a direct neural connection to the brain via the vagus nerve (Moradi et al., 2021), which does not cross the gut epithelium to interact directly with the GM but instead transmits gut information via enteroendocrine cell receptors that bind to synapses in the gut branches of the vagus nerve. Vagus nerve receptors transmit signals to the CNS by sensing regulatory gut peptides, inflammatory molecules, dietary components, and flora metabolites (Borre et al., 2014; Bravo et al., 2011). Thus, the vagus nerve may signal upward to induce an anti-inflammatory response early in gut infections and before circulating cytokines are elevated (Kaelberer et al., 2018; Wang and Powley, 2007; Siopi et al., 2023). Some GMs can directly activate neurons, such as *Lactobacillus rhamnosus*, *B. fragilis*, and *polysaccharide A* isolated from *B. fragilis*, which have all been shown to activate gut afferent neurons directly (Mao et al., 2013).

Goehler et al. (2005), in order to verify whether localized gut infection rapidly activates the vagus nerve, orally inoculated *Campylobacter jejuni* into a mouse model and found that c-fos expression was increased in the brainstem vagus sensory ganglia and primary sensory relay nuclei. In contrast, there was no increase in the level of pro-inflammatory cytokines in the circulatory system. This suggests that the GM interacts with enteroendocrine cell receptors by releasing neurotransmitters via the vagal synapses to transmit signals to neurons and rapidly modulate CNS nerve cell excitability. This finding provides theoretical solid support for the treatment of epilepsy and other neurological diseases by modulating GM. Vagus nerve stimulation has become an accepted treatment for epilepsy since it was first reported in 1988 (Attenello et al., 2015). Ressler and Mayberg (2007) found that electrical stimulation of vagal afferent fibers altered the concentrations of 5-HT, *γ*-aminobutyric acid (GABA), and glutamate in the brain, thereby reducing seizure duration, frequency, and severity.

The microbiota may also affect the CNS by altering adult hippocampal neurogenesis (AHN) (Lin et al., 2016). The adult hippocampus and lateral ventricles function to produce new neurons. The AHN has a vital role in learning and memory and that can influence the pathogenesis of many neurological disorder-related diseases and symptoms, such as epilepsy, depression, Alzheimer’s disease, and Parkinson’s disease. It was found (Atmaca et al., 2017) that there were differences in hippocampal neurons between germ-free (GF) and normal mice and that colonization of the colony after weaning did not alter the number of AHN, suggesting that microorganisms play an essential role in AHN early in life.

### 2.2 Endocrine pathway

There are more than 20 types of enteroendocrine cells in the gut, constituting the largest endocrine organ in the body (Gunawardene et al., 2011). The endocrine pathway is mediated by the enteroendocrine signaling pathway (Latorre et al., 2016) and the enterochromaffin cell signaling pathway (Xu et al., 2021). Enteroendocrine cells have chemosensory mechanisms that initiate appropriate functional responses through gut-brain and brain-gut interactions, some of which involve direct enteroendocrine cell-neuron connections through basal cytoplasmic processes (Psichas et al., 2015). The primary mechanism by which enteroendocrine cells communicate with nerves, either directly or indirectly, is through the release of peptides that act in the intestinal mucosa (Dockray, 2013; Furness et al., 2013). It plays a key role in the control of gastrointestinal secretion and peristalsis, regulation of food intake, postprandial blood glucose levels and metabolism (Latorre et al., 2016). Enterochromaffin cells are the most abundant subtype of colonic enteroendocrine cells, and although they make up less than 1% of intestinal epithelial cells, they produce and release 90% of the body’s serotonin, which is essential for intestinal motility, platelet function, immune response, and bone development (Karsenty and Yadav, 2011; Matthes and Bader, 2018). The enteroendocrine signaling pathway is mainly composed of enteroendocrine cells and enterochromaffin cells. Enteroendocrine cells receive stimuli and then influence CNS function in an endocrine and paracrine manner. Endocrine transmission is mainly mediated by enteroendocrine cells, which convert afferent signals into chemical stimuli by interacting with intestinal microbial metabolites, such as short chain fatty acids (SCFAs) (Tolhurst et al., 2012), secondary bile acids (Joyce and O’Malley, 2022), and tryptophan metabolites (O’Mahony et al., 2015), to induce the gut secretion of neurohormones, chemotransmitters, and other informational molecules and their effects on the hypothalamus and its related brain regions through the circulatory system (Lum et al., 2020). An essential component of endocrine transmission is the Hypothalamic–pituitary–adrenal (HPA) axis. When subjected to stress, the HAP axis releases cortisol, which regulates the activity of gut immune cells and the release of cytokines (Tetel et al., 2018). Moreover, cytokine release regulates the osmotic function and barrier function of the gut and the structure of GM (Mayer, 2000). At the same time, GM can also regulate the HPA axis to affect brain activity (Mangiola et al., 2016). Furlano et al. (2001) reported for the first time that symbiotic gut microorganisms are associated with the HPA axis, which is vital for learning and memory, and that disruption of the HPA axis can lead to hippocampal memory impairment. GF mice have reduced levels of brain-derived neurotrophic factor, N-methyl-D-aspartate (NMDA), and c-fos, which play a role in memory retention, cognition, and other brain functions (Bravo et al., 2011; Rusch et al., 2023).

Paracrine transmission is mainly mediated by the enterochromaffin signaling pathway, which consists mainly of enterochromaffin cells and GM. GM can produce a series of neurotransmitters beneficial or detrimental to the CNS (Tran and Mohajeri, 2021), according to changes in the gut milieu during digestion and metabolism. They also affect CNS activity by acting on the vagus nerve. For example, *Lysinibacillus fusiformis* produces SCFAs and secondary bile acids, which increase tryptophan utilization and stimulate enterochromaffin cells to synthesize human serotonin (5-Hydroxytryptamine, 5-HT) and release it into the circulation. When there is too much or too little 5-HT in the circulation, enterochromaffin cells communicate with central vagal afferent nerve fibers through synaptic connections between neuropod-like extensions and enteric vagal afferent nerve endings to regulate the synthesis and release of 5-HT and to maintain a stable level of 5-HT in the circulation (Ridaura and Belkaid, 2015). Some GM secrete neurotransmitters independently, such as *γ*-aminobutyric acid (GABA) secreted by *Bifidobacterium*, which can directly cross the gut barrier and the BBB to enter the CNS (Braga et al., 2024; Takanaga et al., 2001). When GM structure is altered, it may mediate pro-excitatory effects of peripheral inflammation by activating the immune system through the release of cytokines such as: tumor necrosis factor-alpha (TNF-α) and monocyte chemoattractant protein-1, chemokines and lipopolysaccharides (LPS) (Erny et al., 2015). It is also involved in epileptogenesis by regulating the excitatory and inhibitory neuronal balance through the production of neurotransmitters, especially glutamate, GABA and 5-HT (Paiva et al., 2024).

### 2.3 Immune pathway

The immune pathway is mediated by GM (Fung et al., 2017), which influences neurophysiological processes such as neurodevelopment, neurotransmission, CNS immune activation, and BBB integrity by regulating the development and function of CNS immune cells (microglia and astrocytes) (Liu et al., 2024; Ding et al., 2022). GM also induces inflammation in the brain by influencing the immune system of the gut mucosa and mediating peripheral immune responses, which can lead to brain damage and abnormal behavior (Mu et al., 2022).

Astrocytes and microglia also play an essential role in regulating immune responses in the CNS. Astrocytes are the most abundant glial cells in the brain and are involved in neurotransmitter recycling, immune response, and regulation of BBB integrity. Microglia are macrophages in the brain that promote inflammation and participate in immune monitoring (Aloisi, 2001). Interactions between astrocytes and microglia lead to pro-inflammatory cytokine production and increased BBB permeability, which allows infiltration of peripheral blood immune cells and cytokines into the CNS and leads to chronic neuroinflammation, which is involved in epilepsy pathogenesis (Moradi et al., 2021; Devinsky et al., 2013).

In addition to glial cells in the CNS, GM affects brain function through three main immune pathways (De Timary et al., 2017): Cytokines induced by GMs to be produced enter the circulatory system and enter the brain through the transporter system on the BBB, which has a direct effect on brain activity and function; Toll-like receptors (TLRs) are also expressed on macrophages cells in the circumventricular organs and choroid plexus, which respond to microbe-associated model molecules (MAMPs) of GM in the circulatory system and release cytokines. Since the circumventricular organs is outside the BBB, the released cytokines enter the brain by free diffusion and affect brain activity; IL-1 receptors expressed by perivascular macrophages and epithelial cells of small brain vessels can bind directly to IL-1 produced by GM in the circulatory system, generating prostaglandin E2 that regulates brain activity and function.

The immune mechanism plays a vital role in the onset and development of epilepsy, and the pathogenesis of some refractory epilepsy patients is closely related to the immune mechanism (Gozubatik-Celik et al., 2017; Maranduba et al., 2015). In a meta-analysis of epilepsy and systemic autoimmune diseases, Lin et al. (2016) found that patients with epilepsy had a more than 2.5-fold increased risk of developing autoimmune diseases, and patients with autoimmune diseases had a more than 2.5-fold increased risk of epilepsy, suggesting that epilepsy and autoimmune diseases often co-occur and that aberrant immune responses are involved in epilepsy pathogenesis. Atmaca et al. (2017) investigated the prevalence of neuronal autoantibodies in 22 patients with persistent status epilepticus of unknown cause. The study’s results showed that the overall prevalence of antibody-positive patients was 22.7% (5/22). This suggests that neuronal autoantibodies are involved in the pathogenesis of immunologic factors in epilepsy.

These studies demonstrate that GM can modulate host susceptibility to epileptic seizures through immune mechanisms. Therefore, immune modulation and GM reconstruction may be important strategies for treating epilepsy in the future.

### 2.4 Substance metabolism pathways

GM stimulates the body to produce a variety of neurotransmitters and modulators, cytokines, and metabolites (Bienenstock et al., 2015), such as 5-HT, dopamine, melatonin, GABA, histamine, acetylcholine, IL-1, and SCFAs, which not only have a direct effect on the enteric nervous system and the vagus nervous system, but also act on the enteroendocrine cells and affect the activities of the CNS through the endocrine and paracrine effects. For example, *spore anaerobic bacteria* can regulate the synthesis and secretion of 5-HT by enterochromaffin cells (Bauer et al., 1963). Disruption of this complex homeostatic relationship between the MGB axis affects the regulation of CNS functions such as mood, behavior, memory, and cognitive function and can lead to various CNS disorders (De Timary et al., 2017; Cerdo et al., 2017).

GM induces neurophysiological changes in the host by producing chemicals that bind to receptors inside and outside the gut (Manolios et al., 1988). Compared with specific pathogen free (SPF) animals, GF animals lacking GM colonization showed significant changes in neurobiochemical indicators and related behaviors. For example, GF mice had significantly lower serum levels of 5-HT, defective learning memory, and more “risk-taking behaviors.” After the colonization of the intestines of GF animals with the microbiota of normal animals, serum and colonic levels of 5-HT, adrenocorticotropic hormone, and corticosterone reached normal levels, suggesting that the HPA axis is a critical structure in GM regulation affecting the stress response and the development of the CNS (Furlano et al., 2001; Torrente et al., 2002).

SCFAs, including acetic acid, propionic acid, and butyric acid, are produced by GM through the fermentation of insoluble dietary fibers (Den Besten et al., 2013). SCFAs play essential roles in microglia maturation, enteric and brain nervous system, BBB permeability, and stress response through direct or indirect pathways, and all are closely related to epilepsy (de Caro et al., 2019). Propionic acid reduces seizure intensity and prolongs seizure latency by reducing mitochondrial damage, inhibiting hippocampal apoptosis, and ameliorating neurological deficits (Cheng et al., 2019). In animal models, SCFAs can affect neurodevelopmental and cognitive functions in animals with neurodegenerative diseases (Erny et al., 2015). Aloisi (2001) injected SCFAs into the blood of rats and showed that serum levels of propionic acid were significantly higher (*p* < 0.05) than in normal controls, and neurochemical changes such as neuroinflammation, increased oxidative stress and depletion of antioxidants were observed, resulting in mitochondrial dysfunction. Neural activation molecules serotonin, melatonin, histamine, acetylcholine, and other microbial sources also play a role in the MGB axis (Devinsky et al., 2013).

### 2.5 Relationship between double barrier in upward pathway and epilepsy

Epilepsy occurs as a result of disruption of brain homeostasis and overactivation of neurons by stimuli such as inflammatory factors. The gut barrier and the BBB protect brain homeostasis mainly in the MGB axis (Braniste et al., 2014). The integrity of both barriers is associated with GM. GM protects the integrity of the gut barrier (Martin et al., 2018) and maintains gut homeostasis (Hooper and Macpherson, 2010; Renz et al., 2011; Stecher and Hardt, 2011) by regulating cell growth, differentiation, and promoting the expression of occluding (OCLN) and claudin 5 (CLDN 5), so that peripheral inflammatory factors and neurotransmitters are unable to enter the bloodstream, thus maintaining the BBB integrity and ensuring the CNS homeostasis (Braniste et al., 2014).

#### 2.5.1 Gut barrier

Based on composition and function, the gut barrier is categorized into biological, chemical, physical, and immune barriers (Mowat, 2003). Among them, gut barrier permeability is an indicator reflecting the integrity of the gut barrier (Usuda et al., 2021). The increase of some pathogenic GM and its metabolites, such as *Fusobacteria*, *Verrucomicrobia*, and *Nitrospirae*, induces inflammation, degrades mucins, and increases nitrite toxicity (Engevik et al., 2021; Mao et al., 2021), which further reduces the stability of the gut mucus layer and the integrity of the gut epithelium, and the increase in gut barrier permeability, triggering a “leaky gut” (Usuda et al., 2021). Signaling molecules, such as cytokines, chemokines and neurotransmitters, are transmitted from the gut lumen to the lamina propria or the blood circulation, increasing the permeability of the BBB and leading to “leaky brain” (Logsdon et al., 2018), triggering epilepsy.

##### 2.5.1.1 Gut biobarrier and epilepsy

The gut is the most significant interface between the human body and the external environment, and the GM it contains forms a significant biological barrier for the body (Lavelle and Sokol, 2020). A healthy and intact biobarrier with *anaerobic Bacteroidetes* and *Firmicutes* accounting for more than 90% of the total number of bacterial species, with the remaining facultative bacilli being *Proteobacteria* and *Actinobacteria*, prevents the onset of neuropsychiatric disorders such as multiple sclerosis (iMSMS Consortium, 2022), autism (Xiong et al., 2016), Parkinson’s disease (Sampson et al., 2016) and epilepsy (Ding et al., 2022).

Chronic physical and psychological stress can lead to structural and functional changes in GM that predispose to epileptogenesis. When rats are subjected to chronic restraint stress, the number of pro-inflammatory flora associated with *Lysinibacillus fusiformis*, *Helicobacter pylori*, *Peptococcaceae*, *Streptococcus*, and *Enterococcus faecalis* is elevated, and susceptibility to epilepsy is increased. A small amount of electrical stimulation of the basolateral amygdala induces complete and sustained epileptic seizures in rats. Further, rat GM was transplanted into the gut of SPF rats, and SPF rats transplanted with GM had reduced thresholds for seizures induced by electrical stimulation, whereas SPF rats receiving GM from pseudo chronic restraint stress rats had seizure thresholds and seizure durations as in the control group (Medel-Matus et al., 2018).

A chronic lifestyle of a high-fat, high-sugar, high-carbon-water diet can induce obesity, leading to an increase in the abundance of the *Firmicutes* and a decrease in the abundance of the *Bacteroidetes*, which disrupts the GM structure and lowers the seizure threshold (Bell, 2015). Kang et al. (2015) found that high-fat diet-induced obese mice treated with kainic acid ignition exhibited more grade IV or V seizures on the behavioral Racine classification, a significant increase in the number of seizure spikes and slow waves and seizure duration on the electroencephalogram, and an increase in pathologic hippocampal neuronal death. However, the ketogenic diet (KD), which is high in fat, adequate in protein, and low in carbohydrates, has beneficial effects on many neurological disorders, including Alzheimer’s disease (Phillips et al., 2021), Parkinson’s disease (Jiang et al., 2023), and epilepsy (Pizzo et al., 2022). Ketones may confer neurologic protection and have antioxidant, anti-inflammatory, cellular, epigenetic, and gut microbiome modifying effects (Simeone et al., 2018; Zhang et al., 2018; Boison, 2017). It has been shown that children with refractory epilepsy have a reduced *α*-diversity of intestinal flora, a significant increase in the abundance of *Bacteroides*, and a decrease in the abundance of *Firmicutes* and *Actinobacteria* after treatment with KD, and that the increase in *Bacteroides* may be closely related to the antiepileptic effect of KD by modulating the secretion of IL-6 and IL-17 in dendritic cells (Zhang et al., 2018; Xie et al., 2017). The KD decreases the biodiversity of the gut microbiota and alters the proportions of specific microbiota (e.g., *Akkermansia* and *Parabacteroides*), which increases *γ*-glutamyl amino acids levels, increases epilepsy susceptibility and affects the levels of other gut microbe-derived metabolites, such as SCFAs, lactic acid (Xie et al., 2017; Prast-Nielsen, 2023; Gubert et al., 2020).

Antibiotic use is one of the significant factors contributing to GM dysregulation. Short-term administration of antibiotics significantly targets and alters GM composition, neurogenesis, apoptosis, and synaptic modifications (Ianiro et al., 2016; Jakobsson et al., 2010). Long-term high doses of both *β*-lactams (i.e., penicillins, cephalosporins, and carbapenems) and quinolones have been shown to inhibit GABA synthesis or block GABA receptors (Segev et al., 1988; Hantson et al., 1999). At the same time, taking carbapenems and quinolones can reduce the glutaminergic N-methyl-D-aspartate (NMDA) receptor agonist activity, which is more likely to stimulate excitatory transmission and lower the seizure threshold (Lode, 1999; Wanleenuwat et al., 2020; Schmuck et al., 1998).

##### 2.5.1.2 Gut chemical barrier and epilepsy

The enterochemical barrier consists of a mucus layer and neurotransmitters metabolized by microorganisms. The mucus layer comprises gut epithelial cells with bacteriostatic substances secreted by the GM, which facilitate gastrointestinal transit and separate bacteria from epithelial cells in the gut to maintain gut homeostasis (Cai et al., 2020). When the mucus layer is disrupted, pathogenic microbiota attaches and invades the mucus layer, causing gut diseases (Kudelka et al., 2020; Song et al., 2023). Gut diseases will further aggravate the dysbiosis and increase the number of pathogenic bacteria, which will produce more LPS to induce epithelial cell damage and exacerbate the destruction of the mucus layer, thus triggering “leaky gut,” which will cause LPS to escape into the blood circulation and stimulate the synthesis of pro-inflammatory factors by glial cells of the CNS, such as interleukin-1β and tumor necrosis factor, and increase neuronal excitation, which will lead to a rise in neuronal excitability (Usuda et al., 2021; Mazarati et al., 2021). Johansson et al. (2014) found an impenetrable mucus layer in the gut of healthy people. At the same time, bacteria were detected on the gut epithelial cells of both animal models and patients with colitis.

##### 2.5.1.3 Gut physical barrier and epilepsy

The gut physical barrier consists mainly of gut epithelial cells and intercellular tight junction proteins (Chelakkot et al., 2018). The gut epithelial cells not only form a physical barrier between microorganisms and the lamina propria, but also play an essential role in gut immunity by recognizing pathogenic bacteria and their components (Goto and Ivanov, 2013) (e.g., LPS). The epithelial cells are connected by tight junction proteins such as OCLN and CLDN 5. When the structure of gut epithelium is abnormal, or the intercellular junctions are disrupted, the physical barrier of the gut is impaired, and the gut mucosal permeability increases, which leads to the dysbiosis of the gut microbiota by bacterial translocation and causes diseases. Gut inflammation is associated with increased expression of anti-claudin 2 antibody (claudin-2) and decreased expression of tight junction protein (Weber et al., 2008). Alhasson et al. (2017) found that LPS-induced changes in GM led to intestinal damage, resulting in increased claudin-2 expression, decreased OCLN expression, increased intestinal mucosal permeability, and transfer of LPS from the intestine to the bloodstream. At the same time, the significant increase in TLR-4 expression and the increased expression of inflammatory bacteria such as *Dorea* spp., *Proteus* spp., *Coprococcus* spp., *Ruminococcus* spp., and *Turibacter* spp. led to an increase in the secretion of the inflammatory factor IL-1β and monocyte chemoattractant protein-1, and these toxins and inflammatory factors stimulated the BBB via the blood circulation, causing its endothelial cells to secrete proteases (e.g., matrix metalloproteinase), lipase, and carbohydrase into the extracellular matrix, disrupting BBB zonula occludens protein 1, OCLN, and CLDN 5, and further increasing the permeability of the BBB, which facilitates the entry of pathogens and inflammatory factors (e.g., LPS, Aβ, IL-1β, and IFN-*γ*) from the bloodstream into the brain parenchyma, which then induces neuroinflammation and ultimately causes seizures (Welcome, 2019).

##### 2.5.1.4 Gut immune barrier and epilepsy

More than half of the body’s immune cells are in the gut mucosa (Tlaskalova-Hogenova et al., 2005). The gut immune barrier consists mainly of gut mucosal lymphoid tissue and is distributed within the gut or scattered in the gut epithelium and lamina propria. GM affects the number and distribution of immune cells (Gao et al., 2018). For example, GF mice have incompletely developed lymphoid structures, reduced levels of T and B cells, and reduced cytokine production (Bauer et al., 1963; Manolios et al., 1988); Autistic patients with gastrointestinal disorders had a higher number of infiltrating T cells as well as a higher distribution of CD19^+^ B cells in the duodenal, terminal ileum, and colon biopsy tissues compared to non-inflammatory controls (Furlano et al., 2001; Torrente et al., 2002; Ashwood et al., 2003). In addition, GM diversity is critical for microglia maturation and CNS function; for example, mice treated with sterility and antibiotics produce defects in microglia morphology and maturation, activation, and differentiation, leading to deficient immune responses to a wide range of pathogens, which are later restored to normal by colony transplantation (Erny et al., 2015). GM modulates innate immunity, adaptive immunity, and inflammatory mechanisms to control the development of epilepsy.

GM induces epilepsy through the innate immune pathway. Microglia are macrophages in the CNS that mediate innate immune responses (Ginhoux et al., 2013), promote inflammation, modulate neuronal activity, clear neurons and induce chronic seizures. Microglia of GF and antibiotic pretreated mice were morphologically and functionally immature and had defective immune responses to a wide range of pathogens, but immune responses returned to normal after recolonization with healthy flora (Erny et al., 2015). Peptidoglycan, which is mainly found in human GM, is an essential component of the bacterial cell wall and activates mainly microglia in GM-to-brain signaling (Spielbauer et al., 2024), promoting “leaky gut” and “leaky brain” through the induction of inflammatory cytokines (e.g., interleukin IL-1β, IL-18),” which allows the entry of gut-associated metabolites such as peptidoglycan to drive chronic inflammation in the CNS, leading to hippocampal sclerosis and the induction of temporal lobe epilepsy (Gales and Prayson, 2017).

GM induces epilepsy through the adaptive immune pathway.GM induces immune cells to produce cytokines that enter the brain through the gut barrier and the BBB and activate brain immune cells to participate in the immune response. T helper cell 17 (Th17) is a pro-inflammatory CD4^+^ T cell subset that is a key component of adaptive immunity (Chow and Mazmanian, 2009). Interleukin 17 (IL17) is a cytokine produced by Th17 cells and is regulated by specific GMs such as *Bacteroides vulgatus* group (Ivanov et al., 2008). It has been found that patients with epilepsy have higher levels of IL-17 in the cerebrospinal fluid and peripheral blood than controls, which is highly correlated with the frequency and severity of seizures (Han et al., 2020). Thus, GM influences epileptogenesis by mediating IL17 production from Th17. GM metabolites, such as SCFAs, influence immunoglobulin synthesis and secretion by regulating B lymphocyte differentiation (Honda and Littman, 2016; Kim et al., 2016). In the face of exogenous infections, the organism secretes IgA to protect intestinal epithelial cells from infection by commensal flora, and immunoscreens commensal flora to maintain intestinal immune homeostasis (Fagarasan et al., 2010; Sonnenberg et al., 2012); Meanwhile, GM and flora metabolites stimulation induces gut dendritic cells and primitive B cells to secrete protective IgA, which regulates the development and function of immune cells in the body (Yu et al., 2018). Deficiency of gut commensal flora down-regulates IgA and IgG expression and up-regulates IgE expression, increasing epilepsy susceptibility (Gensollen et al., 2016; McCoy et al., 2017).

GM mediates CNS inflammation to induce epilepsy (Li et al., 2021). GM is necessary for microglia maturation and astrocyte activation. Vascular endothelial growth factor-B and transforming growth factor-*α* promote pathogenic and inflammatory responses in astrocytes (Rothhammer et al., 2018). GM metabolizes dietary tryptophan into an aromatic hydrocarbon receptor agonist and interacts with microglia receptors to activate microglia, upregulate the expression of vascular endothelial growth factor-B and transform growth factor-alpha, and modulate the pathogenic response activity of astrocytes (Rothhammer et al., 2016). Meanwhile, inflammatory factors released by astrocytes increase microglia activity, including migration, phagocytosis, and synaptic modifications in apoptotic cells (Yang et al., 2020). Interactions between astrocytes and microglia result in increased inflammatory response, increased BBB permeability, and easier infiltration of peripheral blood immune cells and cytokines through the BBB into the CNS, causing chronic neuroinflammation to occur and increasing susceptibility to epilepsy (Moradi et al., 2021).

#### 2.5.2 Blood–brain barrier

The blood–brain barrier is an active interface between the systemic circulation and the CNS that maintains homeostasis within the brain by blocking the entry of potentially toxic or harmful substances and regulating the transport of nutrients and the removal of metabolites.

GM has a key role in regulating blood–brain barrier permeability (Xie et al., 2023). Michel and Prat (2016) found lower expression of tight junction proteins and higher BBB permeability in GF mice compared to SPF mice. When transplanting GM from SPF mice or GM that can produce short-chain fatty acids, the expression of tight junction proteins is elevated and BBB permeability is normalized. Stress, inflammation or drugs disrupt the GM structure, and pathogenic bacteria such as *Firmicutes*, *Clostridium* and *Proteusbacillus Vulgaris* increase the LPS content of the gut, activating TLR-4 to release large amounts of cytokines and trigger “leaky gut,” which leads to the entry of cytokines into the blood circulation (Obrenovich, 2018); At the same time, under the influence of pathogenic bacteria, the expression of gut tight junction proteins decreases and gut permeability increases, which allows cytokines TNF-*α* and IL1-*β* to enter the body circulation (Ait-Belgnaoui et al., 2012), and cytokines entering the body circulation through the two pathways work together to disrupt the expression of vascular cell adhesion molecule 1 and intercellular adhesion molecule 1 in the BBB, which increases the permeability of the BBB, and induces “brain leakage,” cytokines and peripheral immune cells enter the brain inducing microglia and astrocytes to activate in large numbers, releasing cytokines, inducing neuroinflammation and contributing to epileptogenesis (Moradi et al., 2021). At the same time, the increased extracellular levels of potassium and glutamate due to the destruction of the BBB increase the excitability of the brain’s neurons, causing a sustained state of abnormal excitation in the brain’s neural network, leading to seizures (Thion et al., 2018).

## 3 Effects of epilepsy on GM via the downward pathway

**Figure 3:**
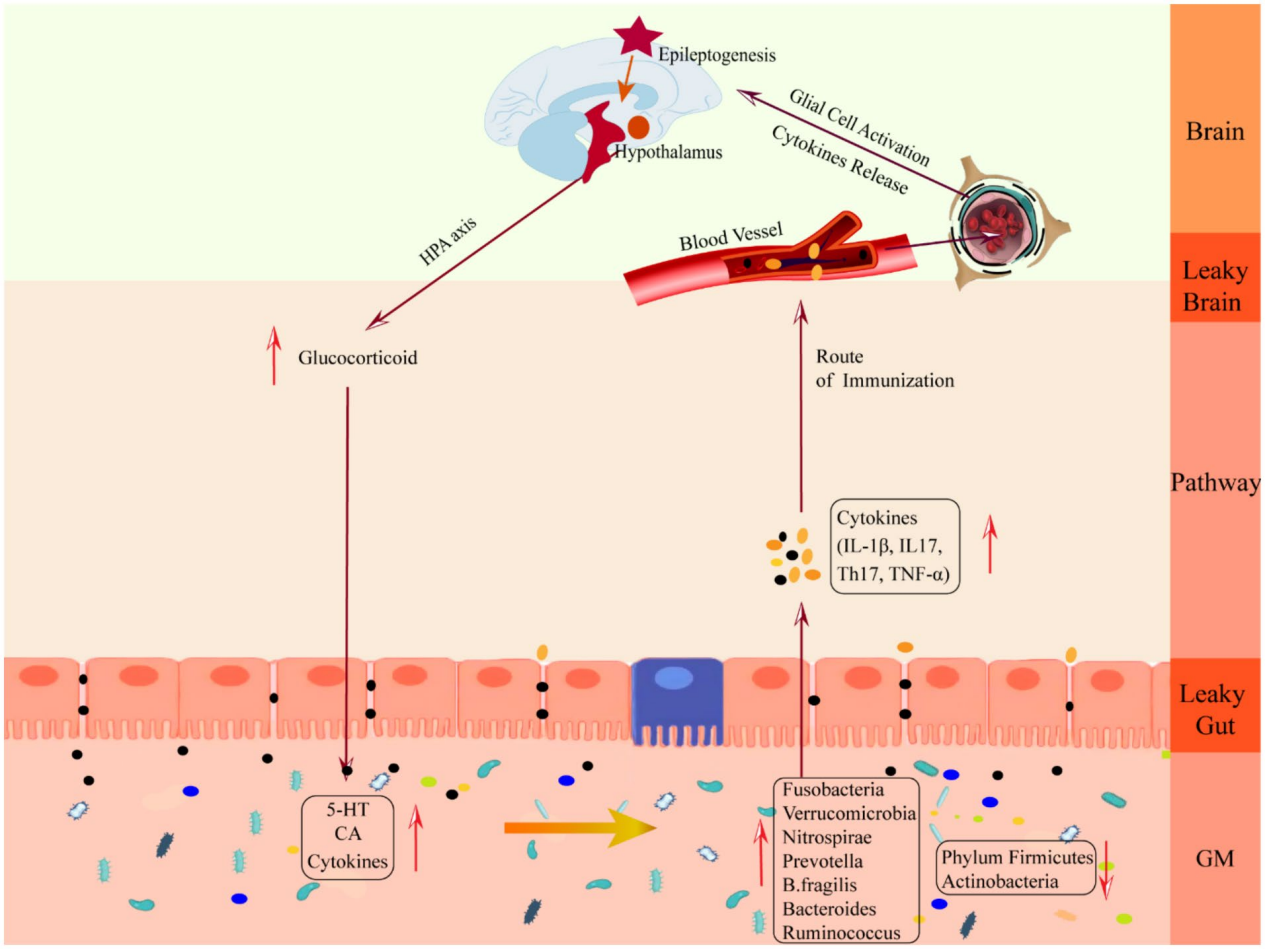
Schematic diagram of the mechanism of damage in the downward pathway “epileptic brain-gut-microbiota”. CA, catecholamine; GM, gut microbiota; IL-1β, Interleukin-1β; IL17, Interleukin 17, TH17, T helper 17; TNF-*α*, tumor necrosis factor-α.

The downward pathway mainly proceeds through the direct neural and neuroendocrine pathways (see Figure 3). The direct neural pathway is mediated by two branches of the autonomic nervous system. It induces changes in intestinal physiology by regulating intestinal functions such as intestinal motility, gastric acid secretion, gut barrier permeability, and mucosal immune responses, which regulate GM composition and activity (Bonaz et al., 2018). The CNS mediates the neuroendocrine pathway. It communicates directly with the GM by activating the HPA axis or releasing neurotransmitters from neurons, immune cells, and enterochromaffin cells, such as catecholamines, cytokines, and 5-HT (Martin et al., 2018).

Clinical studies have shown that patients with CNS injury are often accompanied by severe gastrointestinal dysfunction, mainly manifested by inhibition of gastrointestinal peristalsis and damage to the gastrointestinal mucosa (Iruthayarajah et al., 2018; Camara-Lemarroy et al., 2014), which leads to changes in the gut microenvironment, such as disturbances in the gut immune system (Huh and Veiga-Fernandes, 2020) and translocation of gut bacteria (Stanley et al., 2016), resulting in increased gut permeability and ultimately changes in the structure of the gut flora (Singh et al., 2016). At the same time, inflammatory factors produced by interactions between gut neurons and immune cells, as well as secretions and metabolites of the gut flora, can enter the circulation through the gut barrier, exacerbating peripheral inflammation and CNS damage (Durgan et al., 2019; Liu et al., 2019; Arneth, 2018).

### 3.1 Mechanisms by which epilepsy alters GM through the autonomic nervous system

Autonomic nerves regulate gut physiology, environment and GM composition and activity through the enteric nervous system to maintain host gut homeostasis (Rubio et al., 2023). Following epileptogenesis, sympathetic and parasympathetic nerves (vagus nerves) are activated in response to inflammatory factor stimulation, releasing neurotransmitters, such as catecholamines and acetylcholine, into the peripheral circulation, where they act on gut sympathetic and parasympathetic ganglia (see Figure 2B).

There are two subpopulations of gut sympathetic postganglionic nerves, a subpopulation consisting of vasoconstrictor neurons, secretory inhibitory neurons, and motor inhibitory neurons, which slow gastrointestinal secretion and motility by inhibiting cholinergic transmission and attenuating smooth muscle stimulation. Diminished gut motility decreases gut water content, nutrient absorption and bacterial clearance and increases gut transit time. Gut transport time is strongly correlated with microbial abundance and composition (Vandeputte et al., 2016), and increased gut transport time reduces bacterial biomass and diversity in the distal gut region (Tottey et al., 2017). Reduced bacterial clearance prevents the timely expulsion of harmful flora from the gut, allowing them to adhere to the surface of gut cells (Jänig, 2006); another subpopulation is involved in mucosal immunity by interacting with the GM, the gut mucosa. Epileptogenic bacteria stimulate the gut by releasing endotoxins or substances such as SCFAs and acetylcholine, which reduce gut blood flow, causing apoptosis of gut epithelial cells and reduction of cupped cells. Meanwhile, harmful bacteria attached to the surface of gut cells bind to molecules on the surface of gut epithelial cells, disrupting the expression of tight junction proteins and increasing the permeability of the gut mucosa (Mracsko et al., 2014; Shi et al., 2018).

Gastrointestinal parasympathetic nerves consist of the vagus and sacral parasympathetic nerves, and the vagus nerve directly stimulates the gastric wall cells to secrete acid. It indirectly stimulates the gastric sinus G-cells to release gastrin, which increases the concentration of H^+^ ions to promote the overproduction of histamine and increase gastrointestinal permeability (Li et al., 2020). At the same time, the vagus nerve increases gut barrier permeability via enteric nerves and/or cells (e.g., enteric neuroglia) (Bonaz, 2022). Increased gut barrier permeability triggers a “leaky gut,” which causes bacterial translocation and an inflammatory response, disrupting the gut environment and microbial survival conditions and altering the characteristics of the microflora (Elenkov et al., 2000).

### 3.2 Mechanisms by which epilepsy alters GM via the HPA axis

The HPA axis is regulated by the prefrontal cortex, hippocampus, and amygdala, which are at the core of the stress response and one of the key facilitators of epilepsy induction (Cano-Lopez and Gonzalez-Bono, 2019). When activated, neurons in the paraventricular nucleus of the hypothalamus synthesize and secrete adrenocorticotropin-releasing hormone (Ashwood et al., 2013), which causes the anterior pituitary gland to release adrenocorticotropic hormone through the pituitary portal system, which stimulates the adrenal cortex to synthesize and secrete glucocorticoid hormones and to enter the blood circulation, binds to the glucocorticoid receptor in the gut tract or in the brain to exert its effects, and acts on the negative feedback loop between the hypothalamus and the pituitary gland to regulate the secretion of the HPA axis (Smith and Vale, 2006). The brain can influence gut homeostasis by regulating changes in the HPA axis. GABA is an inhibitory neurotransmitter in the brain. When seizures occur, GABAergic control of hypothalamic paraventricular nucleus neurons, which are at the apex of HPA axis control, is impaired (O’Toole et al., 2014), the negative feedback loop is disrupted, and the HPA axis is overexcited, resulting in massive production and release of glucocorticoids, leading to increased cerebral excitability (Castro et al., 2012) and human cortisolism (Tetel et al., 2018), gut immune cells, gut epithelial cells and enteroendocrine cells are over-activated, and large amounts of epinephrine and norepinephrine stimulate the GM sensing mechanism, increasing the toxicity of several intestinal pathogens as well as non-pathogenic microorganisms, which collectively trigger an gut immune-inflammatory response and decrease gut barrier permeability. At the same time, excessive glucocorticoids increase gut barrier permeability (Moussaoui et al., 2014), reduce motility, and decrease mucus secretion (Da et al., 2014), which triggers “leaky gut,” which translocates harmful bacteria (Tena-Garitaonaindia et al., 2022) and breaks down the GM structure, triggering gastrointestinal disorders such as abdominal pain, colitis, or irritable bowel syndrome (Park et al., 2013), which is a mechanism of gastrointestinal dysfunction in epileptic patients. Mechanism of gastrointestinal dysfunction in patients with epilepsy.

## 4 Characterization of GM changes in patients with epilepsy

Differences in microbial composition exist between patients with epilepsy and healthy individuals. These differences are due to a variety of factors, such as inflammatory responses, altered neurotransmitters, and medication. These factors can affect the gut environment and microbial conditions and alter the characteristics of the microflora.

Patients with epilepsy have an abundance of harmful bacterial phyla such as *Fusobacteria*, *Verrucomicrobia*, and *Nitrospirae* in their gut, and a lower number of *Firmicutes* and *Spirochaetes*. There is a positive correlation between *Fusobacteria* and serum 2-Deoxy-D-ribose levels (Liang et al., 2020). 2-Deoxy-D-ribose together with thymidine phosphorylase and vascular endothelial growth factor inhibit tight junction proteins leading to the disruption of the BBB and causing inflammatory disorders of the CNS (Chapouly et al., 2015). Meanwhile, 2-Deoxy-D-ribose promotes seizures by inducing hippocampal neuronal apoptosis through oxidative stress (Formichi et al., 2009). *Verrucomicrobia* produces high abundance of SCFAs that degrade mucins and increase the permeability of the BBB and small intestinal epithelial barrier by altering OCLN and CLDN 5 expression (De la Cuesta-Zuluaga et al., 2017). *Verrucomicrobia* are positively correlated with the serum pro-inflammatory cytokine IFN-*γ* (Lin et al., 2019), and with altered permeability, bacterial translocation and neuroinflammatory substances are susceptible to enter the brain and induce epilepsy (Gao et al., 2017). *Verrucomicrobia* is involved in epileptogenesis by increasing glutamate and glutamine levels and decreasing serotonin levels (Sun et al., 2016). *Nitrospirae* increases nitrite toxicity, which ultimately leads to structural disruption of the BBB with increased permeability and triggers epilepsy. In patients with chronic inflammatory diseases, the abundance of *Spirochaetes* is much lower than in healthy individuals (Cao et al., 2018), suggesting that *Spirochaetes* plays a protective role in immune defense.

Patients with epilepsy have elevated levels of *Prevotella*. *Prevotella* can produce SCFAs and when in high abundance can lead to sustained production of IL-6 in the gut, triggering an inflammatory response, and neuroinflammation can lead to neuronal hyperexcitability, causing seizures (Sanz and Garcia-Gimeno, 2020). *Prevotella* is involved in seizures by affecting cytokine levels, but the mechanism of action needs to be further elucidated. *B. fragilis* is also closely associated with epilepsy. *B. fragilis* is an anaerobic rod-shaped *Gram-negative* bacterium that secretes pro-inflammatory substances, such as LPS and *B. fragilis* toxins, which cause an increase in the permeability of the BBB through E-calmodulin in epithelial cells, making it easier for microbial-derived neurotoxic substances to enter the brain and promote neuronal hyperexcitability that triggers epilepsy (Lukiw et al., 2021; Vezzani et al., 2016). Thus, *B. fragilis* and its epileptogenic metabolites induce a neuroinflammatory response by disrupting the BBB.

Epilepsy-associated microflora dysbiosis can also be characterized by changes in the abundance of certain genera. In epileptic patients the abundance of the genera *Bacteroidetes* and *Ruminococcus* is increased. Colonization of *Ruminococcus* in GF mice resulted in increased levels of the intestinal pro-inflammatory cell Th17, and activation of T17 increased the risk of autoimmune epilepsy (Han et al., 2020). *Ruminococcus* has been shown to be strongly associated with irritable bowel syndrome (Hall et al., 2017), Crohn’s disease (Nagayama et al., 2020) and generalized anxiety (Jiang et al., 2018), among others. *Rumatococcus* promote epilepsy progression by exacerbating inflammation and inducing intestinal mucosal degeneration (Clark and Mach, 2016). Furthermore, the gut microbiome of patients with drug-resistant epilepsy differs from that of patients with drug-sensitive epilepsy. Drug-resistant patients have increased alpha diversity and relative abundance of thick-walled phyla (Peng et al., 2018).

In conclusion, significant differences in GM composition were observed in patients with epilepsy compared to healthy individuals. *Prevotella*, *Bacteroides*, *Fusobacterium*, *B. fragilis*, *Ruminococcus*, and *Nitrospirae* are potential risk factors for seizures, and elevated levels of *Clostridium* and *Verrucomicrobia* can be used as a biomarker for the diagnosis of epilepsy (Dong et al., 2022). Characteristic changes in GM and metabolic pathways in epileptic patients suggest new targets for the treatment of epilepsy.

## 5 Summary and outlook

In summary, the present study reveals the association between GM and epilepsy based on the upward and downward pathways of the MGB axis. Gut dysfunction/disorders are closely associated with the susceptibility and pathogenesis of epilepsy, and dysregulation of GM leads to increased gut barrier and BBB permeability, which induces neuroinflammatory substances to enter the brain, leading to increased neuronal excitability and inducing epileptic seizures. Frequent seizures repeatedly activate intracranial immune response, excessive activation of the autonomic nervous system and HPA and other downward pathways, triggering “leaky brain “, causing inflammatory factors to be transported to the gut through the blood circulation, destroying the gut barrier and triggering “leaky gut,” leading to bacterial translocation; at the same time, gut sympathetic nerve excitation inhibits gastrointestinal motility, causing excessive accumulation of harmful bacteria and their metabolites, which in turn increases susceptibility to seizures through the upward pathway. At the same time, gut sympathetic nerve excitation inhibits gastrointestinal peristalsis, leading to excessive accumulation of harmful bacteria and their metabolites, which alters the structure and function of the GM, which in turn increases the susceptibility to seizures through the upward pathway. If not intervened, breaking the “positive feedback” MGB axis loop will increase the frequency and duration of seizures. There are differences in microbiome composition between epileptic patients and healthy individuals, and these differences are due to inflammatory responses, neurotransmitter alterations, medications, and other factors. Some specific GM may serve as gut biomarkers and potential therapeutic targets in patients with epilepsy. By regulating GM it may be possible to improve the symptoms and prognosis of patients with epilepsy. In conclusion, dysregulation of GM is a potential risk factor for causing epileptic seizures, and epileptogenic GM and its metabolites become biomarkers for diagnosing epilepsy after seizures; the characteristic changes in GM and metabolic pathways may provide new targets for epilepsy treatment.

In the future, the following aspects can be improved: (1) Further investigate the mechanism of action between GM and epilepsy, including the effect of microbial metabolites on neuronal excitability, and the regulation mechanism of gut barrier permeability. (2) Compare the microbial composition and functional changes in patients with different types of epilepsy, and search for biomarkers of specific microbiota to provide a basis for the diagnosis and treatment of epilepsy. (3) To explore the therapeutic effects of precise gut microbial agents, such as probiotics and prebiotics, on epilepsy, and to provide new strategies for the individualized treatment of epilepsy.
